# Archaeomagnetic dating as a tool to overcome the Hallstatt plateau: A combined chronological approach at the salt production site of Piscina Torta (Rome, Italy)

**DOI:** 10.1371/journal.pone.0351625

**Published:** 2026-07-08

**Authors:** Luca Alessandri, Anita Di Chiara, Raquel Bonilla-Alba, Luca Cusimano, Giovanni Alberto Della Sala, Angelica Fiorillo, Virginia Gianni, Caterina Rossi, Agostino Sotgia, Fabio Florindo

**Affiliations:** 1 University of Groningen, Groningen Institute of Archaeology, Groningen, Netherlands; 2 University of Rome La Sapienza, Department of Science of Antiquity, Rome, Italy; 3 Istituto Nazionale di Geofisica e Vulcanologia, Rome, Italy; 4 University of Rome Tor Vergata, Department of History, Culture and Society, Rome, Italy; 5 Alma Mater Studiorum, Università di Bologna, Department of Cultural Heritage, Ravenna, Italy; University of Mazandaran, IRAN, ISLAMIC REPUBLIC OF

## Abstract

The salt production site of Piscina Torta (Rome, Italy) presents a complex chronological challenge due to its ceramic assemblage, which is almost exclusively represented by coarse ware jars with limited morphological variability. Furthermore, its occupation period coincides with the Hallstatt plateau, which hampers radiocarbon dating resolution. This study addresses these limitations by applying archaeomagnetic analyses to a kiln and ceramic fragments both from Area 1 of the site. Archaeomagnetic dating based on directions from the kiln and on archaeointensity from pottery sherds yielded two-time intervals: 930–755 BCE and 740–700 BCE. These results confirm the potential of this method as a viable dating tool, especially for problematic radiocarbon periods. At Piscina Torta, the integration of archaeomagnetic, typo- chronological, and radiocarbon data enabled the subdivision of Area 1 into three occupational phases: Phase A (725–710 BCE), Phase B (710–550 BCE), and Phase C (550–525 BCE). This study demonstrates the effectiveness of archaeomagnetism in enhancing chronological resolution in early first-millennium BCE contexts and highlights its important contribution when combined with traditional dating methods.

## 1. Introduction

### 1.1. The archaeological site of Piscina Torta

**Fig 1:**
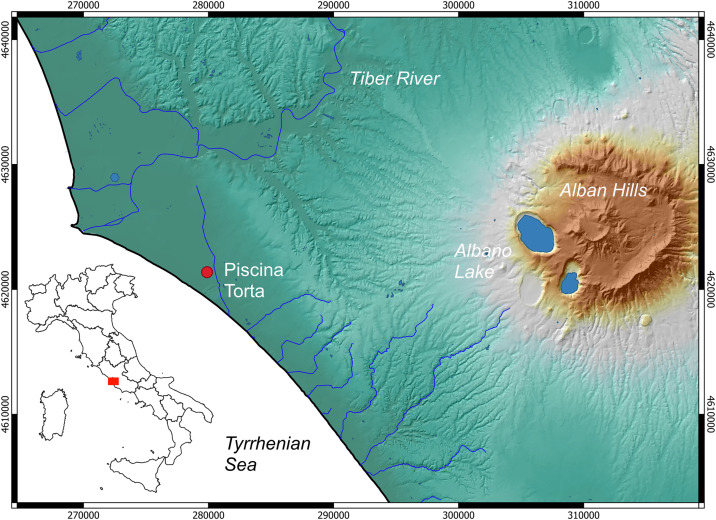
Positioning of Piscina Torta archaeological site (EPSG: 32633). Background, elevation data from SRTM DEM (CC BY 4.0).

The site of Piscina Torta [1] encompass a minimum area of approximately 20 hectares. It belongs to a category of specialized settlements widely distributed along the central Tyrrhenian coast [2]. These sites share certain common characteristics, the most evident of which is the almost exclusive presence within the ceramic record of large quantities of fragmented coarse ware (impasto) jars, typically reddish in colour. These specialised settlements are commonly interpreted as centres of salt production, where brine was artificially evaporated inside these jars through a technique known as briquetage [3–5]. Since 2022, Piscina Torta has been the focus of multiple excavation campaigns, aimed at examining its archaeological record [6] (Fig 1), conducted within the framework of the Salt and Power project at the University of Groningen, in collaboration with Sapienza University of Rome and the University of Rome Tor Vergata [7,8].

The materials examined in this paper originate from Area 1, an excavation area opened in 2022 following preliminary survey campaigns, with the purpose of identifying kilns, either for ceramic production or for salt processing.

The excavation campaigns constrained the lifespan of Area 1 at Piscina Torta into three phases. The earliest (Phase A) corresponds to the period of active use of the area, almost certainly for salt production, though not necessarily limited to that activity. This can be inferred from the presence of several so-called pedestals, which are consistently associated with salt production in European contexts [4]. In the second phase (Phase B), the area changes its function and becomes a dumping zone, both for materials resulting from similar production processes carried out nearby, and likely also for the soil removed during dredging operations of the adjacent canal. Finally, in the third phase (Phase C), Area 1 is definitively abandoned, although this may have occurred before the complete abandonment of the site as a whole. We therefore define Phase C as the possible interval between the abandonment of Area 1 and the abandonment of the entire Piscina Torta site. The kiln 1K3 and the SU 138 belong to Phase A. S II belongs to Phase A and Phase B. The radiocarbon dates were obtained from stratigraphic units (SU 115 and SU 122) assigned to Phase B. The surface finds, for the reasons mentioned above, are representative of the final phases of the entire Piscina Torta site and must therefore encompass, within their time span, at least Phase B of Area 1.

### 1.2. The chronological issues

Intensive surface surveys, conducted prior to the excavation, have provided a chronological framework for the site based on ceramic comparisons, dating it between 675 and 525 BCE [6,9]. Notably, the area has never been subjected to plowing, ensuring the preservation of deeper stratigraphic layers. This factor raised the possibility that the site’s occupation chronology may extend further back in time. However, the fact that almost all of protohistoric ceramic fragments recovered from the site (excluding evidence of late republican and imperial Roman occupation, which is beyond the scope of this study) were identified as jars has posed challenges for chronological assessment. Jars constitute a ceramic category characterized by minimal morphological variability over time, which has traditionally led excavation reports to prioritize more diagnostically significant forms, such as cups and bowls. Consequently, jars have been systematically underrepresented in the archaeological record, further complicating the identification of suitable comparative material and thus impeding reliable and precise chronological determinations.

**Fig 2:**
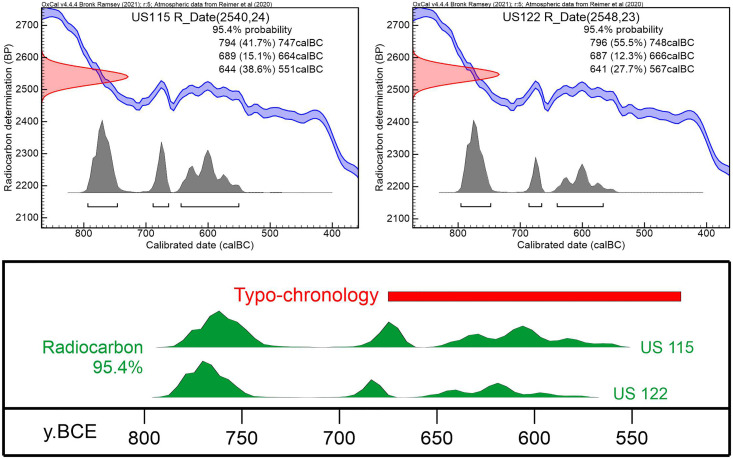
Chronological constraints from Piscina Torta.

In the case of Piscina Torta, the chronological situation is further complicated by the difficulty in obtaining radiocarbon dates that are sufficiently precise or comparable to typo-chronological assessments. This complication arises because the site’s occupation period falls within the so-called Hallstatt Plateau [10–12], a segment of the calibration curve (ca. 800−400 BCE) characterized by an extended almost flat region. Within this plateau, radiocarbon calibration typically yields very broad and/or multimodal date ranges, which does not improve the dating accuracy obtained with typological criteria. The only two radiocarbon determinations obtained thus far from Piscina Torta, from animal bones, (Table 1) encompass after calibration an average interval of approximately 235 years. This age interval nearly corresponds to the entire lifespan of the settlement as established by the ceramic typo-chronological criteria (Fig 2), therefore not improving the dating accuracy. In order to obtain a more refined chronological constrain we turn to an alternative dating tool based on archaeomagnetism.

**Table 1:** Radiocarbon dates from Piscina Torta. Sample prov., sample provenance; Lab. ref., unique laboratory reference code; ^14^C date, expressed in years BP (Before Present, where present is conventionally fixed at 1950 CE); 1σ (sigma), uncertainty at 68.2% confidence; CalBC, calibrated age interval obtained using OxCal 4.4.4 and the IntCal20 curve, at 95.4% confidence [13].

Sample prov.	Material	Lab. ref.	^14^C date	± 1σ	CalBC
Area 1, SU 115	Ovis Aries, tibia	GrM-32621	2540 BP	24	794−551 (95.4%)
Area 1, SU 122	Fauna indet, rib	GrM-32687	2548 BP	23	796−567 (95.4%)

### 1.3. The archaeomagnetic dating

The aim of this study is to obtain archaeomagnetic dating from both a kiln and unoriented pottery sherds. First, through the application of archaeomagnetic analysis to one of the investigated kilns (kiln 1K3) we aim at obtaining an absolute date may serve to challenge, confirm, or refine the internal chronology of the site and specifically, that of the excavation area designated as Area 1. Second, through the archaeointensity investigation of some ceramic samples we aim at obtaining an archaeomagnetic dating, without the need for in situ sampling, offering an additional age constrain to the Area 1 chronology, and exploring the possibility that this approach could represent a valid alternative to radiocarbon dating at this site.

Archaeomagnetism, the investigation of Earth’s magnetic field (EMF) variations recorded in archaeological materials [14,15], can provide invaluable and independent information on archaeological chronologies [16,17]. From direct observations (satellites) and indirect data (palaeomagnetic records) we understand that the EMF constantly changed in time (in decadal, century, millennial scales and beyond), both in its direction (declination -the angle with the geographical North- and inclination -the angle with the horizontal plane) and strength (intensity). Archaeological materials which were subjected to high temperatures (above ca. 580° C, the Curie Temperature of magnetite) during their making and/or use acquire a thermal remanent magnetization (TRM) recording the EMF at the time of firing. Hence, archaeomagnetic dating is based on the comparison between the TRM values recorded by our undated object with EMF variations predicted from paleoreconstructions (global or regional geomagnetic models or paleosecular variation reference curves) (Gallet, 2021). Four requirements need to be fulfilled for a feasible archaeomagnetic dating study: i. a detailed paleomagnetic reconstruction of the EMF as a local paleosecular variation curve or a regional geomagnetic model need to be available for the studied region; ii. the EMF needs to be characterised by significant and relatively fast variations in time (e.g., a hyperactive period in the studied region); iii. the archaeological material needs to be characterised by magnetic properties which ensure a stable behaviour in recording a reliable TRM [e.g., 18, 19]; and iv. the archaeological material needs to have been reached temperatures above the Curie point of the ferromagnetic minerals within the archaeological material to record a TRM.

For the area of central Italy, the first two requirements are satisfied as we can rely on both a regional paleosecular variation curve [20,21] and the robust regional model based on European TRM data SCHA.DIF.4k for the last 4 millennia [22]. Moreover, during the expected period of occupation of Piscina Torta site, the EMF was characterised by a remarkable feature, recording extremely high strength and rapid variations, named the Levantine Iron Age Anomaly (LIAA) [23,24]. First recognized in archaeomagnetic data from Syria [25] and confirmed in several other studies from ancient Mesopotamia [26]; it has been reported also in further areas, such as in Central Europe [20,27].The LIAA is a period of extremely high intensities, with at least two spikes (the 10^th^ century BCE and then during the 8^th^ c. BCE [28]). These rapid variations and high intensity peaks allow a high resolution archaeomagnetic dating.

To fulfil the other two requirements, we have tested the rock magnetic stability of the analysed materials available for this study and evaluated the firing conditions at the Piscina Torta.

Finally, archaeomagnetic dating can be performed by using ArchaeoPyDating, an open source python-based online tool developed by Serrano et al. [17], based on the Matlab archaeo_dating tool of Pavón‐Carrasco et al. [29]. This online tool allows to compare the obtained data of declination, inclination and/or intensity with several reference curves at the site location and provides probability density functions of ages’ distribution.

The archaeomagnetic dating was carried out on a kiln located in Area 1 (1K3) using the archaeomagnetic directions on two pillars, while on a selection of potsherds retrieved from the deepest stratigraphic layers of the sector, namely Sounding II (S II) and stratigraphic unit 138 (SU 138), we based the archaeomagnetic dating on archaeointensity only.

## 2. Materials and methods

### 2.1. The kiln 1K3

**Fig 3:**
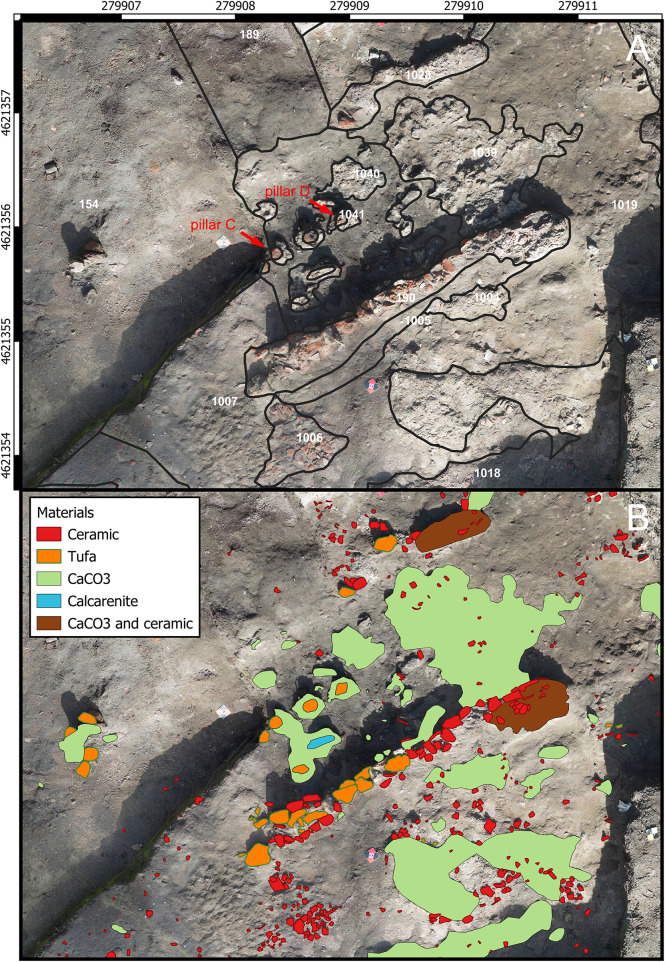
Thee kiln 1K3 in Area 1. A, orthomosaic showing the stratigraphic units found in association with the kiln. The red arrows indicate the sampled pillars; B, characterization of the recovered materials.

**Fig 4:**
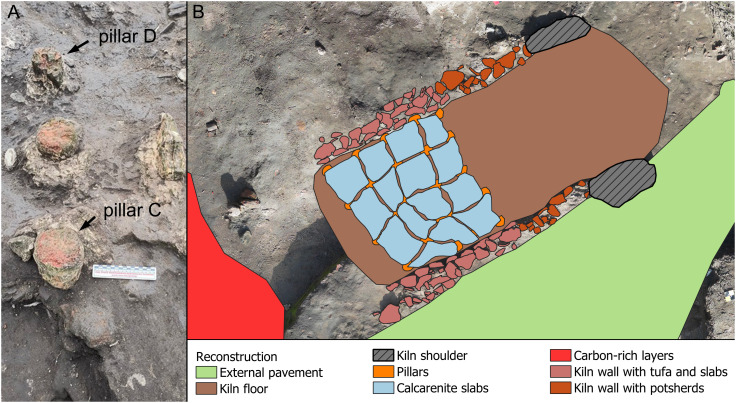
A, Picture of the Pillar C (closer to the supposed fire) and D (farthest to the supposed fire) sampled for archaeomagnetic analyses; B, possible reconstruction of the original appearance of the kiln. The calcarenite slabs forming the raised floor likely covered the entire surface of the structure (they are shown here only on one half for illustrative purposes).

Kiln 1K3 is located in the northern part of Area 1 (Fig 3). It is a rectangular structure measuring approximately 320 x 180 cm, oriented from northeast to southwest. The structure is preserved almost entirely along its southern wall, with part of the northern wall still visible, as well as portions of two successive floors made of calcium carbonate. Some small tuff pillars embedded in the flooring, which once supported a raised floor set around 15 cm above the floor, are also preserved. Two of them have been sampled for archaeomagnetic dating (Pillar C and D, Figs 3 and 4). No materials that could be interpreted as kiln covering were found during the excavation and no burnt layers were discovered inside the kiln; however, they are present in large quantities in the westernmost part of the sector, near the southwest opening of the kiln (Fig 4). The interpretation of the kiln—whether it was used for salt production, ceramics, or foodstuffs—remains complex and falls outside the scope of this contribution.

### 2.2. Stratigraphic Unit 138

In the stratigraphic sequence of Area 1, SU 138 is one of the deepest stratigraphic units, in direct contact with the undisturbed soil. For comparison, the two SUs dated using radiocarbon analysis are positioned significantly higher in the stratigraphic sequence.

SU 138 is a compact sandy-matrix layer with a dark brown-black coloration, characterized by the presence of numerous ceramic fragments, charcoal, tuff inclusions, limestone clasts, and a single bronze ring. In terms of the ceramic assemblage, on the surface fragments measuring between 2 and 6 cm account for approximately 20% of the total area, while those ranging from 6 to 20 cm constitute around 40%. Most of the recovered ceramics belong to impasto jars, although a number of decorated impasto potsherds are also present, along with a fragment of a Euboean skyphos.

### 2.3. Sounding II

Sounding II (S II) extends down to the sterile soil. It consists of a trench with approximate dimensions of 1x6m, oriented roughly NE-SW. Within the trench, alternating centimetre-thick layers were identified, consisting of accumulations of ceramic fragments and calcium carbonate deposits.

The ceramic-rich layers have been interpreted as refuse deposits derived from productive activities carried out at the site, most likely associated with salt production. The extensive calcium carbonate deposits may result either from the preliminary processing of salt, given that CaCO₃ is the first of the compounds to precipitate during brine boiling [8,30], or from the systematic dredging of the (partially) artificial canal located a few meters from the site, which connected the sea with the lagoon situated behind the settlement.

### 2.4. The potsherds from SU 138 and S II

**Fig 5:**
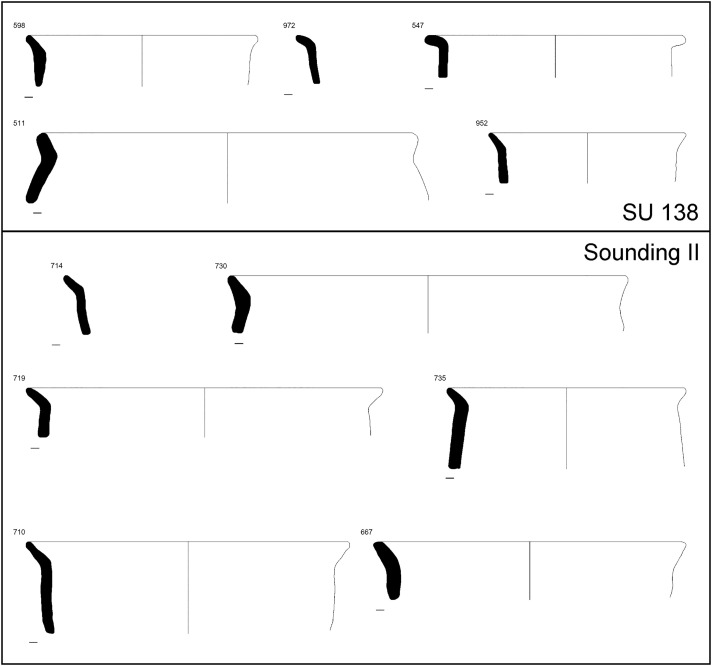
Ceramic fragments from SU 138 and Sounding II dated using typological analysis and archaeomagnetic dating (in the figure the prefix PT has been omitted in front of the inventory number).

Eleven potsherds from SU 138 (five potsherds) and S II (six potsherds) were selected primarily based on their state of preservation to enable the most precise possible typological dating, thereby providing an independent chronological control (Table 2). All the fragments belong to jars with a surface colour between brown and reddish (Fig 5). The preliminary chronological assessment of the ceramic fragments has been established through comparative analysis with analogous specimens from previously dated contexts, selected for their proximity to the site of Piscina Torta.

**Table 2:** Potsherd parallels and chronology. ID, potsherd code; SU, stratigraphic unit; RMCA, Roma Colli Albani facies.

ID	SU	Ceramic parallels	Chronology of the ceramic parallels (BCE)
**PT952**	138 (up)	Satricum, Stratum IA [31] n.1587)	750−725 (end of RMCA III)
**PT972**	138 (up)	Satricum, Stratum IIB ([31] n.2271); Ficana ([32] fig. 119, 33b)	630−600; 760−570 (with 64% of specimens in 760−690)
**PT511**	138	Satricum, Stratum IA ([31] n.1565)	750−725 (end of RMCA III)
**PT547**	138	Crustumerium, sito E ([33] tav. XXV,4); Ficana, Fossa (site A), SU12/16 ([34] fig. 32, 31)	Orientalizing or archaic phase; RMCA IV (A + B)
**PT598**	138	Palatino, Via Nova-Via Sacra, att. 7,8,10,16,28 [35] type 28);	730/20-700; 700−675; 650-630/20; 550-530/20
**PT667**	Sounding II	Satricum, E9 hut 5 ([31] n. 1016)	700−600
**PT710**	Sounding II	Fidene ([36] fig. 8,3)	800−750
**PT714**	Sounding II	Fidene ([32] fig. 119, 34a)	760−600 (with 86% of specimens in 760−690)
**PT719**	Sounding II	Ficana, zona 4a, T XIII ([37] fig. 2);; Ficana ([32] fig. 122, 36c); Palatino, Via Nova-Via Sacra, att. 53 ([35] type 406); Satricum, square D3 ([31] n. 142); Satricum D10, hut II ([31] n. 591)	RMCA IVA; 760−570 (with 61% of specimens in 760−690); 530/20–500; Iron Age and Archaic (disturbed layer); 770–750/40;
**PT730**	Sounding II	Palatino, Via Nova-Via Sacra, att. 52 ([35] type 434); Satricum, square C4 ([31] n.9); Satricum, Stratum IA ([31] n.1570); Satricum, square E10, hut I ([31] n. 349)	RMCA IV; 830−725 (RMCA IIB-III); 750−725 (end of RMCA III); 830−750
**PT735**	Sounding II	Palatino, Via Nova-Via Sacra, att. 52 ([35] type 408); Crustumerium, sito G ([33] tav. XXVI,11); Ficana ([32] fig. 120, 35a)	530/20–500; Orientalizing or archaic phase; 760−570 (with 62% of specimens in 760−690)

### 2.5. Materials and methods for archaeomagnetic study

The archaeomagnetic study was performed on two different sets of materials: the two in-situ pillars of kiln 1K3 and some potsherds from SU 130 and S II. The two pillars, C and D, were collected in situ. Before the extraction from their in-situ position, we capped them with plaster, marking their original orientation with a fiducial mark towards the magnetic North (Figs 3 and 4) for the directional analyses (the compass was corrected from the local declination, but no solar orientation could be obtained). The potsherds (for which no in-situ orientation can be obtained) were collected for archaeointensity measurements.

Rock magnetic experiments were performed to understand the magnetic mineralogy and domains (i.e., their grain size), investigate the nature of the ferromagnetic minerals to evaluate their ability to preserve a characteristic remanent magnetization (ChRM) acquired at the time of the cooling. One temperature-dependent magnetic susceptibility (k-T) curve per pillar was measured using a KLY-5 (AGICO) Kappabridge equipped with a furnace. Each k-T curve was measured up to a maximum temperature of 700◦ C in an air atmosphere, at a medium heating rate of 11.8 ◦C/min and a field intensity of 400 A/m (S1 Fig).

#### 2.5.1. Archaeomagnetic directions.

Standard paleomagnetic cube samples (ca. 8 cm^3^) were cut from the two pillars for archaeomagnetic analyses to obtain archaeodirections. The natural remanent magnetization (NRM) was first measured in 17 samples (13 from pillar C and 4 from pillar D) with a 2G Enterprises DC-SQUID cryogenic magnetometer, hosted in the shielded room of the paleomagnetic laboratory at the National Institute of Geophysics and Volcanology (Rome, Italy). Each sample was then subjected to alternating field (AF) demagnetization cleaning experiments in 16 stepwise demagnetizing steps (from NRM up to 100 mT field).

The demagnetization data were visualized and analysed using PCA analysed on PuffinPlot [38], obtaining orthogonal vector component diagrams from which the magnetization components were isolated by principal component analysis [39]. Site mean archaeomagnetic directions were computed using Fisher statistics [40].

#### 2.5.2. Archaeomagnetic intensities.

From the available potsherds, preliminary thermal demagnetization was performed on 20 pieces (specimens, on one to three per sample) from 10 potsherds (sample) to select the most suitable samples for archaeointensity analyses (S2 Fig). We subjected 10 specimens to routine alternating field (AF) demagnetization in 16 demagnetizing steps (from NRM to 100 mT field) and 10 specimens to a thermal demagnetization (TH) in 7 temperature steps (up to 600 ºC). In the observation of demagnetization results, the selection of samples was restricted to those displaying a single component in their Zijderveld plots, pointing towards the origin.

From these initial results, four samples were selected, PT667, PT547, PT511 e PT719 (Figs 5, S3), and 3–4 specimens of each sample, for a total of 15 specimens, were subjected to the Thellier-Thellier method [15]. All the experiments were conducted in the Paleomagnetism Unit of the CAI Physical Techniques at Complutense University, Madrid (Spain). During the Thellier protocol, specimens were heated and cooled in an applied laboratory field of 50 µT along the Z axis. The temperature was increased progressively in 8 steps from the natural remanent magnetization (NRM) up to 560ºC. Following the methodology described in Bonilla-Alba et al. [41], partial thermoremanent magnetization (pTRM) checks were measured and the thermoremanent magnetization anisotropy correction has been applied. The ratio of the NRM remaining at each step compared to the pTRM gained over the experiment (under the laboratory field) can be assumed to be quasi-linear. This ratio is multiplied by the applied laboratory field, and it is the estimate of the ancient field strength. Archaeointensity analysis was carried out with the StarmacAW3.0 and Stereo_V3.0 software developed by Dr. Pierrick Roperch at Geosciences‐Rennes.

In order to obtain high-quality results, strict selection criteria at both the specimen and sample levels were applied. The specimens utilised for the determination of archaeointensity data satisfy the following criteria: a minimum of five demagnetisation steps (N); the proportion of the TRM employed [f, 42] should exceed 50%. The maximum angular deviation [MAD, 39] should not exceed 5°; the quality factor (q, Coe et al., 1978), a measure of the overall quality of the archaeointensity estimate evaluating the relative scatter of the best-fit line, should be greater than 10; the NRM fraction and the gap factor should exceed 2; and the deviation angle [DANG, 43] should not exceed 5°. To ensure the high quality of the paleointensity determinations, the discrepancy between the original TRMs and the pTRMs for the same temperature should be less than 10%. With regard to the corrections that have been applied, the anisotropy of the TRM was applied at the specimen level (at 500° C in 6 directions, x, y, z, and -x, -y, and -z). Initial archaeointensity results were corrected for cooling rate by decreasing them of 5%, according to the values observed in the archaeomagnetic database by Hervé et al. [44]. The mean archaeointensities at the sample level were calculated from the arithmetic mean of the specimens (after cooling rate and anisotropy corrections), and the error was determined as the standard deviation of the archaeointensities at the specimen level. It should be noted that only samples with a minimum of three accepted specimens were selected for the calculation of mean archaeointensities.

## 3. Results

### 3.1. Typo-chronology of the potsherds

**Fig 6:**
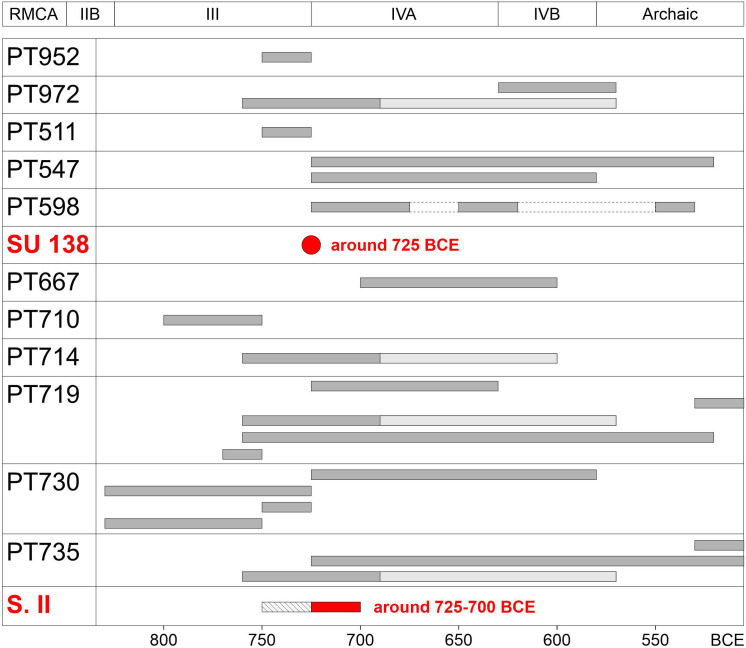
Chronological framework of SU 138 and S II based on typo-chronological analysis. The upper row indicates the Roma-Colli Albani phases, while the lower row shows the corresponding calendar years.

Drawing on the chrono-typological comparisons (Table 2), context SU 138 may be dated to approximately 725 BCE (Fig 6). The fragments from S II, taken as a whole, are datable between 750 and 700 BCE. However, given that S II directly succeeds SU 138 within the stratigraphic sequence, the chronological range can be narrowed to between 725 and 700 BCE. Under this assumption, fragment PT710 would thus represent a residual artifact (Fig 6).

### 3.2. Archaeomagnetic results

#### 3.2.1. Archaeomagnetic directions from pillars and archaeodating.

We analysed 17 oriented specimens from two pillars (Table 3 and Figs 3 and 4) and of these, all four analysed specimens from pillar D gave reliable and well grouped results while only five of the thirteen analysed from pillar C showed consistent and interpretable directions. Interestingly, the mean directions of the two pillars (Table 4) have an angular distance of 3.1°, thus they are statistically indistinguishable. These directions were used for archaeomagnetic dating, which was performed by using ArchaeoPyDating [17] at Piscina Torta coordinates (41.749997° N 12.2999988° E) and the SCHA.DIF.4k regional European model. We tested two approaches to proceed with the archaeodating. The first approach is to treat the two pillars separately, obtaining possible age ranges of 960−675 for Pillar C and 915−775; 720−705 BCE for Pillar D (Table 4 and S4 Fig). These the large time ranges depend on the relatively large uncertainty (angular dispersion at 95%) of the archaeodirections (see Table 4).

**Table 3:** Specimen-level directional results. NRM = Natural Remanent Magnetization, PCA = Principal component analysis, dec. = declination, inc. = inclination and MAD = mean angle deviation.

Specimen	NRM intensity (A/m)	PCA dec. (deg)	PCA inc. (deg)	PCA MAD(º)
**PT_C3**	7.407	29.1	64.5	0.4
**PT_C5**	5.345	37.3	77.9	0.6
**PT_C7**	14.8	21.5	68.5	0.4
**PT_C8**	6.778	36.1	67.6	0.1
**PT_C10**	9.506	22.3	68.7	0.2
**PT_D1**	0.8461	38.9	64.6	0.4
**PT_D2**	0.7635	35.4	68.4	0.2
**PT_D3**	1.538	36.3	65.9	0.2
**PT_D4**	1.389	39.2	64.6	0.2

**Table 4:** Sample-level directional results. n/N = number of successful specimens vs total number of specimens analysed, dec. = declination, inc. = inclination, α_95_ = angular dispersion at 95% confidence..

Site	n/N	Fisher dec. (deg)	Fisher inc. (deg)	Fisher α_95_ (deg)	Fisher k	ArchaeoDating ages (SCHA.DIF.4k)
**pillar C**	5/13	28.6	69.5	5.3	211.0	960−675 BCE
**pillar D**	4/4	37.5	65.9	2.2	1709.8	915−775;720−705 BCE
**pillars**	9/17	33	68	3.1	274	930−755; 740−700 BCE

**Fig 7:**
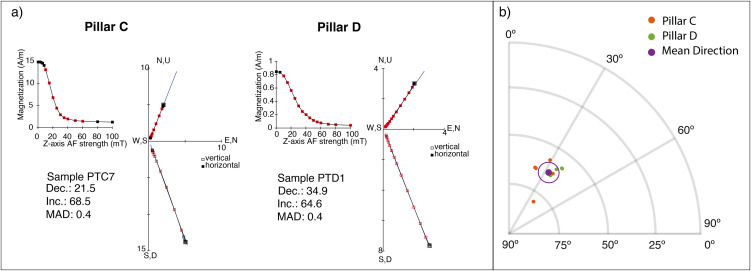
Archaeomagnetic results from Pillar C and D. a) Stereonet showing the mean archaeomagnetic direction for Piscina Torta with two examples of Orthogonal plots, stereonets and magnetization (in A/m) vs field intensity (in mT) plots for two representative specimens from pillar C and D.

**Fig 8:**
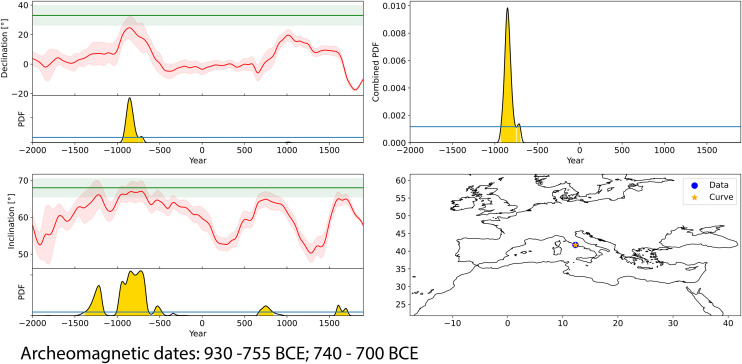
Archaeomagnetic dating of the pillars.

The second approach is by using all specimens from both pillars to calculate the common mean to obtain a dec = 33°; inc = 68° and α_95_ = 3.1° (Fig 7). We obtained two possible age windows, 930−755 BCE and 740−700 BCE (Fig 8).

#### 3.2.2. Archaeomagnetic intensity from ceramics and archaeodating.

**Fig 9:**
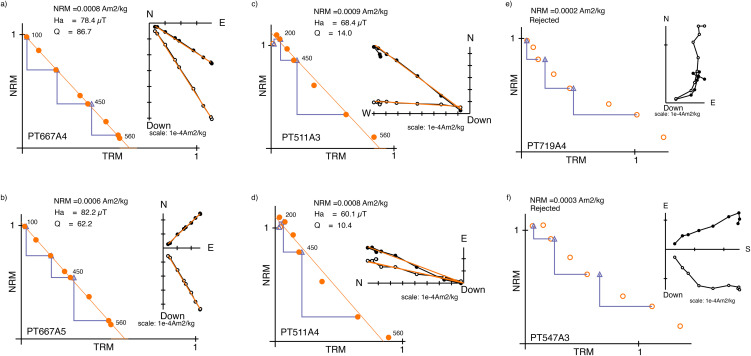
Arai diagrams together with Zijderveld plots for the a-d) accepted and e-f) rejected specimens. The initial natural remanent magnetization (NRM), the archaeointensity obtained after (Ha) the thermoremanent magnetization anisotropy correction and the quality factor (Q).

**Fig 10:**
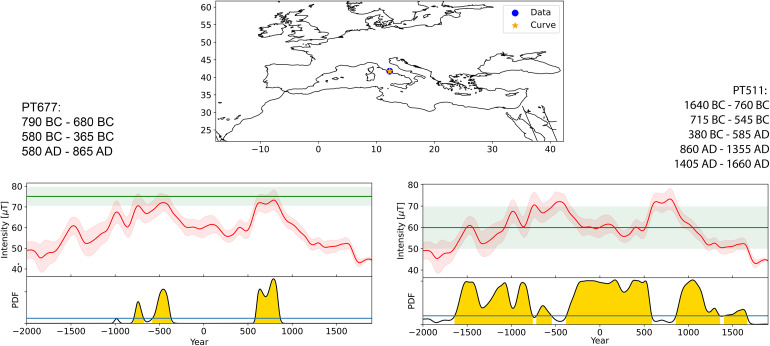
Archaeodating of two ceramics using the SCHA. DIF.4k regional model with the output age.

Of the 15 specimens from four selected ceramic fragments (samples) subjected to the Thellier-Thellier method [15], 9 archaeointensity estimates from two ceramics passed our strict selection criteria (Table 5 column H). In Fig 9 we show some examples of ‘successful’ (Fig 9a-d) and ‘unsuccessful’ (Fig 9e-f) specimens; both examples of unsuccessful specimens show how the pTRM check failed, suggesting an alteration of the magnetic mineralogy during heating. For each archaeointeinsty value from specimens, we applied an anisotropy correction factor, ranging between 0.93 and 1.08 (correction factor, fcor, Table 5). Then, for each sample (ceramic fragment), we calculated the average archaeointensity results, applied the average 5% correction for cooling rate (Table 6) and used these values for archaeomagnetic dating (Fig 10). Sample PT511 gave five possible age ranges, of which only two could be consistent with the site occupation (1640 BC – 760 BC, and 715 BC – 545 BC) while PT667 gave three possible age ranges, two of which (790 BC – 680 BC and 580 BC – 365 BC) are the most reasonable with the archaeological context.

**Table 5:** Specimen-level archaeointensity results. t1-t2 = minimum and maximum temperature steps selected for archaeointensity determination (in °C), N = number of steps analysed, f = the proportion of the TRM employed, q = quality criteria, MAD = maximum angular deviation (in º), DANG = the deviation angle (in º), H = ancient field (in µT), fcor = anisotropy correction factor, and Ha = ancient field (in µT) after the correction.

Specimen	t1	t2	N	f	q	mad	dang	H	fcor	Ha
PT511A3	200	560	7	0.89	14.0	1.7	4.2	69.5	1.08	68.4
PT511A4	200	560	7	0.99	10.4	2.9	1.8	74	0.93	60.1
PT511A5	200	560	7	0.83	8.6	4.4	7.0	69	0.98	60.7
PT667A3	100	560	8	0.75	26.1	4.3	2.9	82.9	0.93	78.6
PT667A4	100	560	8	0.87	86.7	0.9	0.9	82.2	0.95	78.4
PT667A5	100	560	8	0.84	62.2	1.6	0.8	85.2	0.95	82.2
PT667A6	100	560	8	0.80	53.2	1.5	0.8	80.6	0.96	77.1

**Table 6:** Sample-level archaeointensity results corrected for cooling rate. Ha = ancient field (in mT), errHa = standard deviation (in µT), and Dating = archaeodating using the SCHA.DIF.4k regional model (in bold are indicated the most likely age ranges).

Sample	Ha	errHa	Dating
**PT511**	**59.9**	**4.6**	**1640 BC – 760 BC** **715 BC – 545 BC** 380 BC – 585 AD 860 AD – 1355 AD 1405 AD – 1660 AD
**PT667**	**75.1**	**2.2**	**790 BC – 680 BC** **580 BC – 365 BC** 580 AD – 865 AD

## 4. Discussion

**Fig 11:**
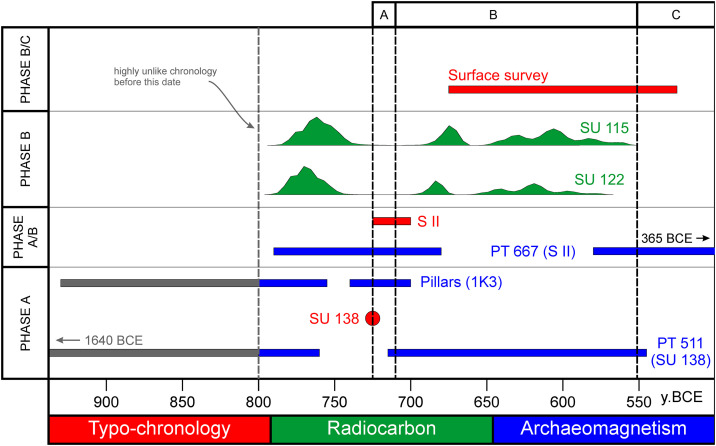
Chronological constraints for the Piscina Torta Area 1 combining archaeomagnetic, radiocarbon and typo-chronological analyses.

As previously noted, from a stratigraphic perspective Area 1 can be divided into three chronological phases (A, B, and C), each corresponding to a distinct use of the area. We will now discuss the chronology of each phase, drawing upon the typological-chronological, archaeomagnetic, and radiocarbon results. It should be preliminarily noted that, from a typo-chronological perspective, it is highly unlikely that the Area 1 predates the 8^th^ century BCE (Fig 11).

### 4.1. Absolute chronology of Area 1

The deepest layers of Area 1 considered here, corresponding to Phase A and coming from SU 138, have been dated to around 725 BCE. The second archaeological interval of the pillars of kiln 1K3 begins in 740 BCE; however, the kiln postdates SU 138, so the earlier date can be retained as the potential beginning of Phase A. The date obtained from S II is consistent with this age. As for the end of Phase A, it cannot predate the beginning of the second archaeomagnetic interval based on fragment PT 511 (once the first interval is excluded), i.e., after 715 BCE. Since S II terminates, on a chrono-typological basis, at 700 BCE, the end of the phase must fall between these two dates. We therefore propose to place Phase A between 725 and around 710 BCE.

The date of 710 BCE as the beginning of Phase B is consistent with both the archaeomagnetic date of PT 667 and the radiocarbon dates from SU 115 (794-551 BCE) and SU 122 (796-567 BCE). As for the end date, the available data are more difficult to interpret. The radiocarbon dates both terminate around or shortly before 550 BCE, which can therefore be regarded as the terminus *ante quem* for the end of Phase B. We therefore propose that Phase B be considered to span from 710 to 550 BCE.

The Phase C should therefore begin in 550 BCE. Since the most recent date obtained from the surface survey is 525 BCE, we propose to define Phase C as spanning from 550 to 525 BCE, interpreted as a phase marking the abandonment of Area 1 prior to the complete abandonment of Piscina Torta.

### 4.2. Archaeomagnetic observations and dating

The archaeomagnetic directions from the two pillars have a discrepancy in terms of robustness of statistics: analysed specimens from Pillar D yielded five out of five reliable results (Table 3 and Figs 3 and 4), statistically more robust and with a higher success rate than those from pillar C (only five of the thirteen). This discrepancy could not be explained from the rock magnetic analyses as both x-T curves show an almost reversible behaviour with a single Tc around 575ºC (Magnetite, S1-S2 Tables). Another possible explanation is by the position of the two pillars with respect to the open fire, with Pillar C being closer to the supposed location of the open fire. When a material is subjected to temperatures higher than the Curie temperature(s) (Tc or if multiple Tcs it is a temperature spectrum) of the magnetic mineral phases contained in the material itself (for instance the Tc of Magnetite is 580°C) it is expected, when cooling below the Tc, to record a TRM with a parallel direction and a proportional intensity of the Earth’s magnetic field by the cooling time. If a previous TRM was present it would have been ‘reset’, removing the previous remanent magnetization. However, if the fire did not reach such high temperatures, a full-TRM would not be acquired. In the case of Piscina Torta, assuming that the 1K3 kiln was used for salt production, the open fire needed to reach only modest temperatures: since the goal is evaporation, it is sufficient for the brine to reach its boiling temperature (this does not exclude the possibility that the kiln temperature could be higher). This observation is consistent with the unusually low success rate in determining a well-defined archaeomagnetic direction for most of Pillar C (only 5 of the 13 analysed specimens are well grouped). On the contrary, Pillar D, located in the farthest position from the open fire, has an archaeomagnetic mean direction well defined, with a 100% success rate, shares a similar archaeodirection with accepted specimens from pillar C.

A possible interpretation is that a higher temperature fire (>600°C) interested at some point the North part of the occupation site for other uses than salt production.

Beyond the possible interpretations of the reasons for the different success rate of the two pillars, an important observation is that the mean directions of the two pillars (Table 4) have an angular distance of 3.1°, thus they are statistically indistinguishable. Hence, the archaeodating has been performed considering a common average direction. The obtained ages ranges of 930−755 BCE and 740−700 BCE (Fig 8) confirm the radiocarbon and the potsherds chronology and it is slightly older than the chronological framework suggested from preliminary surface intensive surveys based on ceramic comparisons, which dated the site between 675 and 525 BCE [6].

Independently, we used the archeointensity results from the two unoriented ceramic fragments (Table 6) for archaeomagnetic dating (Fig 10). Selecting the age intervals consistent with the period of the site occupation, Sample PT511 gave two possible ones, 1640 BC – 760 BC, and 715 BC – 545 BC, and PT667 gave other two possible age ranges, 790 BC – 680 BC and 580 BC – 365 BC. When compared with the morphology ages, archaeomagnetic dating of PT511 seems to agree with the estimated age of 750−725 BCE (end of RMCA III, Table 2), with the most likely age ca. 760 BCE. The archaeomagnetic age interval between 790 BC and 680 BC of sample PT667 would agree with the estimated age of 700−600 BCE (Table 2).

From this study, we highlight the importance of integrating different disciplines. On one side, the radiocarbon and typo-chronological dates helped reduce the uncertainty of the archaeomagnetic dates (and vice versa), significantly contributing to the dating of Area 1 and its subdivision into phases.

On the other side, archaeomagnetism offered a robust and independent dating tool as an alternative to absolute dating techniques, particularly useful for improving archaeological chronologies in periods where traditional dating methods are less effective (e.g., Hallstatt Plateau of radiocarbon dating). In the case of Piscina Torta, archaeomagnetic dates based on magnetic directions allowed for a highly precise dating of kiln 1K3. However, the dates obtained from ceramic fragments, based solely on magnetic intensity, yielded much broader chronological ranges. Even if these cannot help refining the archaeomagnetic dating, it is interesting that we observe high archaeointensity values, consistent with the period of the LIAA.

Finally, increasing the number of samples, and thus the precision, could significantly narrow these ranges refining the archaeomagnetic dating as an independent and robust dating tool and, on the other side, better understand the behaviour of the EMF in the past.

## 5. Conclusion

The analyses aimed at establishing the chronology of Area 1 at the Piscina Torta site have achieved the main objective of obtaining the first highly accurate dating of Area 1 combining different dating methods. According to the new dating, the phases of activity of Area 1 are subdivided in three: Phase A (725–710 BCE), Phase B (710–550 BCE), and Phase C (550–525 BCE).

At Piscina Torta we demonstrated that archaeomagnetic dating can be applied effectively and that the dating effectiveness is enhanced when cross-referenced with dates obtained through other independent methods. Archaeomagnetism represent an effective tool for dating archaeological materials, particularly in periods that are problematic for radiocarbon dating, such as the Hallstatt plateau, and can be used effectively in conjunction with other methods to further narrow chronological ranges.

## Supporting information

S1 Fig Susceptibility-Temperature curves where the heating curve is marked in red and the cooling curve is in blue. (PDF)

S2 Fig Representative thermal demagnetization curves from the studied ceramic collection. (PNG)

S3 Fig Ceramic fragments selected for archaeointensity study. (PNG)

S4 Fig Archaeomagnetic dating of the two pillars ranges. (PNG)

S1 Table Specimen-level archaeomagnetic directional results for pillars C and D of kiln 1K3 is the number of specimens. Data are interpreted using the PuffinPlot Software [38]. PCA = Principal component analysis calculated using the number of demagnetization steps; dec. = Declination; inc. = Inclination and MAD is the Mean Angle dispersion.
(XLSX)

S2 Table Specimen-level archaeointensity determinations obtained with the Thellier-Thellier method (full dataset). Num = specimen name, t1 and t2 are the minimum and maximum temperatures used to calculate the palaeointensity slope; N is the number of steps used for calculations. Hlab is the laboratory field used in the IZZI experiment. H is the ancient field and fcor is the correction factor for the anisotropy correction and Hcorani is the final archaeointensity corrected after cooling rate and anisotropy. All other selection parameters are as in [45].
(XLSX)

## References

[1] Pisani Sartorio G, Quilici Gigli S. Trovamenti arcaici nel territorio laurentino: annotazioni di topografia e prospettive di ricerca. Bullettino della Commissione Archeologica Comunale di Roma. 1984;89:9–26.

[2] Bulian F, Alessandri L, Attema P, Sevink J. Bronze Age to Roman period salt production in the coastal areas of peninsular Italy: Palaeoenvironments, production methods and archaeological evidence. Quat Sci Rev. 2024;344:108930. doi: 10.1016/j.quascirev.2024.108930

[3] Alessandri L. Elite and power in Bronze Age and Early Iron Age Latium vetus. BAR Intern. Latium Vetus in the Bronze Age and Early Iron Age/ Il Latium Vetus nell’età del Bronzo e nella prima età del Ferro. 2013. pp. 17–93.

[4] Harding A. Salt: white gold in early Europe. Cambridge: Cambridge University Press; 2021. doi: 10.1017/9781009038973

[5] Weller O. Aux origines de la production du sel en Europe.Vestiges, fonctions et enjeux archéologiques. Archéologie du sel: techniques et sociétés Colloque 122, XIVe Congrès UISPP. 2002. pp. 163–176.

[6] Alessandri L, Attema PAJ, Bulian F, Sevink J, De Neef W, Baiocchi V, et al. Salt in Late Iron Age Italy. A multidisciplinary approach to the exploration of Italy’s coastal exploitation sites: Piscina Torta (Ostia, Rome) case study. J Archaeol Sci: Rep. 2024;53:104361. doi: 10.1016/j.jasrep.2023.104361

[7] Attema PAJ, Alessandri L, Bulian F, De Neef W, Sevink J. Studying coastal resources and resource control in the context of early state formation on the Tyrrhenian coast (Italy). In: Pirson F, Schütt B, Schulz T, editors. Tagungen und Kongresse 3. 2024. pp. 59–65. doi: 10.34780/b573-qb3b

[8] Alessandri L, Attema P. From briquetage to salterns in protohistoric central Italy. Research of a fundamental subsistence commodity. IpoTESI di Preistoria. 2021;14:161–8. doi: 10.6092/issn.1974-7985/14338

[9] Alessandri L, Baiocchi V, Monti F, Cusimano L, Fiorillo A, Gianni V, et al. Low-cost GPS/GNSS Real Time Kinematic receiver to build a cartographic grid on the ground for an archaeological survey at Piscina Torta (Italy). Acta IMEKO. 2023;12(4):1–6. doi: 10.21014/actaimeko.v12i4.1561

[10] Baillie MGL, Pilcher JR. Some observations on the high-precision calibration of routine dates. In: Ottaway BS, editor. Archaeology, Dendrochronology and the Radiocarbon Calibration Curve. Edinburgh. 1983. pp. 51–63.

[11] Pearson GW, Pilcher JR, Baillie MGL. High-precision 14C measurement of irish Oaks to show the natural 14C variations from 200 BC to 4000 BC. Radiocarbon. 1983;25(2):179–86. doi: 10.1017/s0033822200005464

[12] Stuiver M, Becker B. High-precision decadal calibration of the Radiocarbon Time Scale, AD 1950–2500 BC. Radiocarbon. 1986;28:863–910. doi: 10.1017/S0033822200060185

[13] Reimer PJ, Austin WEN, Bard E, Bayliss A, Blackwell PG, Bronk Ramsey C. The IntCal20 Northern Hemisphere radiocarbon age calibration curve (0–55 cal kBP). Radiocarbon. 2020;62:725–57. doi: 10.1017/RDC.2020.41

[14] Aitken MJ. Dating by archaeomagnetic and thermoluminescent methods. Philos Transac R Soc London Series A, Math Phys Sci. 1970;269(1193):77–88. doi: 10.1098/rsta.1970.0087

[15] Thellier E, Thellier O. Sur l’intensité du champ magnétique terrestre dans le passé historique et géologique. Annales de Geophysique. 1959;15:285–378.

[16] Lanos P. Bayesian Inference of Calibration Curves: Application to Archaeomagnetism. In: Buck CE, Millard AR, editors. Tools for Constructing Chronologies: Crossing Disciplinary Boundaries. London: Springer; 2004. pp. 43–82. doi: 10.1007/978-1-4471-0231-1_3

[17] Serrano M, Pavón‐Carrasco FJ, Campuzano SA, Osete ML. ArchaeoPyDating: A new user‐friendly release for archaeomagnetic dating. Archaeometry. 2024;66(6):1424–37. doi: 10.1111/arcm.13009

[18] Santos CN, Tauxe L. Investigating the accuracy, precision, and cooling rate dependence of laboratory‐acquired thermal remanences during paleointensity experiments. Geochem Geophys Geosyst. 2019;20(1):383–97. doi: 10.1029/2018gc007946

[19] Tauxe L, Santos CN, Cych B, Zhao X, Roberts AP, Nagy L, et al. Understanding nonideal paleointensity recording in igneous rocks: insights from aging experiments on lava samples and the causes and consequences of “fragile” curvature in Arai plots. Geochem Geophys Geosyst. 2021;22(1). doi: 10.1029/2020gc009423

[20] Rivero-Montero M, Gómez-Paccard M, Kondopoulou D, Tema E, Pavón-Carrasco FJ, Aidona E. Geomagnetic field intensity changes in the central Mediterranean between 1500 BCE and 150 CE: implications for the Levantine Iron Age Anomaly evolution. Earth Planet Sci Lett. 2021;557:116732. doi: 10.1016/j.epsl.2020.116732

[21] Tema E, Lanos P. New Italian directional and intensity archaeomagnetic reference curves for the past 3000 years: Insights on secular variation and implications on dating. Archaeometry. 2020;63(2):428–45. doi: 10.1111/arcm.12603

[22] Pavón-Carrasco FJ, Campuzano SA, Rivero-Montero M, Molina-Cardín A, Gómez-Paccard M, Osete ML. SCHA.DIF.4k: 4,000 years of paleomagnetic reconstruction for Europe and its application for dating. J Geophys Res: Solid Earth. 2021;126(e2020JB021237). doi: 10.1029/2020JB021237

[23] Stillinger MD, Hardin JW, Feinberg JM, Blakely JA. Archaeomagnetism as a complementary dating technique to address the iron age chronology debate in the levant. Near Eastern Archaeol. 2016;79(2):90–106. doi: 10.5615/neareastarch.79.2.0090

[24] Vernet E, Carrancho Á, Calvo-Rathert M, Arróniz L, Yamamoto Y, Bógalo MF, et al. Full vector archaeomagnetic dating of an early iron age archaeological settlement: El Castillar site (Navarra, northern Spain). J Archaeol Sci: Rep. 2025;62:105059. doi: 10.1016/j.jasrep.2025.105059

[25] Genevey A, Gallet Y, Margueron J. Eight thousand years of geomagnetic field intensity variations in the eastern Mediterranean. J Geophys Res. 2003;108(B5). doi: 10.1029/2001jb001612

[26] Di Chiara A, Tauxe L, Levy TE, Najjar M, Florindo F, Ben-Yosef E. The strength of the Earth’s magnetic field from Pre-Pottery to Pottery Neolithic, Jordan. Proc Natl Acad Sci U S A. 2021;118(34):e2100995118. doi: 10.1073/pnas.2100995118 34400499 PMC8403961

[27] Hervé G, Faβbinder J, Gilder SA, Metzner-Nebelsick C, Gallet Y, Genevey A, et al. Fast geomagnetic field intensity variations between 1400 and 400 BCE: New archaeointensity data from Germany. Phys Earth Planet Inter. 2017;270:143–56. doi: 10.1016/j.pepi.2017.07.002

[28] Osete ML, Molina-Cardín A, Campuzano SA, Aguilella-Arzo G, Barrachina-Ibañez A, Falomir-Granell F, et al. Two archaeomagnetic intensity maxima and rapid directional variation rates during the Early Iron Age observed at Iberian coordinates. Implications on the evolution of the Levantine Iron Age Anomaly. Earth Planet Sci Lett. 2020;533:116047. doi: 10.1016/j.epsl.2019.116047

[29] Pavón-Carrasco FCOJ, Rodríguez-González J, Osete ML, Torta JM. A Matlab tool for archaeomagnetic dating. J Archaeol Sci. 2011;38(2):408–19. doi: 10.1016/j.jas.2010.09.021

[30] Attema P, Alessandri L, Bulian F, Sevink J, Sotgia A. Production and Demand of Salt in Ancient Italy from the Bronze Age to the Roman Period. In: Eubanks PN, Dumas AA, McKillop H, Alexianu M, editors. Meridians of Salt: Global Perspectives on Archaeology and Ethnoarchaeology. Cham: Springer Nature Switzerland; 2025. pp. 211–233. doi: 10.1007/978-3-031-96692-7_11

[31] Maaskant Kleibrink M. Settlement excavations at Borgo le Ferriere “Satricum.”. Groningen; 1987.

[32] Brandt RJ. Scavi di Ficana. Il periodo protostorico e arcaico. Le zone di scavo 3b-c. Roma: Istituto Poligrafico e Zecca dello Stato; 1996.

[33] Quilici L, Quilici Gigli S. Crustumerium. Roma; 1980.

[34] Fischer-Hansen T, Algreen-Ussing G. Excavations at Ficana III. The Iron Age fortifications. Roma: Edizioni Quasar; 2013.

[35] Carafa P. Officine ceramiche di età regia. Roma: L’Erma di Bretschneider; 1995.

[36] Di Gennaro F, Bartoli F, Foddai E, Giorgietta B, Iaia C, Merlo M, et al. Contesti e materiali della prima età del ferro, di età orientalizzante, arcaica e tardoarcaica da Fidene. 2009 [cited 12 Mar 2025]. Available from: https://www.persee.fr/doc/efr_0223-5099_2009_act_425_1_9815

[37] Jarva E. La funzione della ceramica comune a Ficana: note sulla capacità dei vasi. 2009 [cited 12 Mar 2025]. Available from: https://www.persee.fr/doc/efr_0223-5099_2009_act_425_1_9813

[38] Lurcock PC, Florindo F. New developments in the PuffinPlot paleomagnetic data analysis program. Geochem Geophys Geosyst. 2019;20(11):5578–87. doi: 10.1029/2019gc008537

[39] Kirschvink JL. The least-squares line and plane and the analysis of palaeomagnetic data. Geophys J Int. 1980;62(3):699–718. doi: 10.1111/j.1365-246x.1980.tb02601.x

[40] Fisher R. Dispersion on a sphere. Proc R Soc A: Math Phys Eng Sci. 1953;217(1130):295–305. doi: 10.1098/rspa.1953.0064

[41] Bonilla‐Alba R, Gómez‐Paccard M, Pavón‐Carrasco FJ, del Río J, Beamud E, Martínez‐Ferreras V, et al. Rapid intensity decrease during the second half of the first millennium BCE in Central Asia and global implications. JGR Solid Earth. 2021;126(10). doi: 10.1029/2021jb022011

[42] Coe RS, Grommé S, Mankinen EA. Geomagnetic paleointensities from radiocarbon‐dated lava flows on Hawaii and the question of the Pacific nondipole low. J Geophys Res. 1978;83(B4):1740–56. doi: 10.1029/jb083ib04p01740

[43] Tauxe L, Staudigel H. Strength of the geomagnetic field in the Cretaceous Normal Superchron: New data from submarine basaltic glass of the Troodos Ophiolite. Geochem Geophys Geosyst. 2004;5. doi: 10.1029/2003GC000635

[44] Hervé G, Chauvin A, Lanos P, Rochette P, Perrin M, Perron d’Arc M. Cooling rate effect on thermoremanent magnetization in archaeological baked clays: an experimental study on modern bricks. Geophys J Int. 2019;217: 1413–24. doi: 10.1093/gji/ggz076

[45] Paterson GA, Tauxe L, Biggin AJ, Shaar R, Jonestrask LC. On improving the selection of Thellier-type paleointensity data. Geochem Geophys Geosyst. 2014;15(4):1180–92. doi: 10.1002/2013gc005135

