# Supplementary figures and images for: Archaeomagnetic dating as a tool to overcome the Hallstatt plateau: A combined chronological approach at the salt production site of Piscina Torta (Rome, Italy)
Source: PLoS One. 2026 Jul 8;21(7):e0351625. doi: 10.1371/journal.pone.0351625 (PMC13345286; doi:10.1371/journal.pone.0351625)

a)

PT\_C

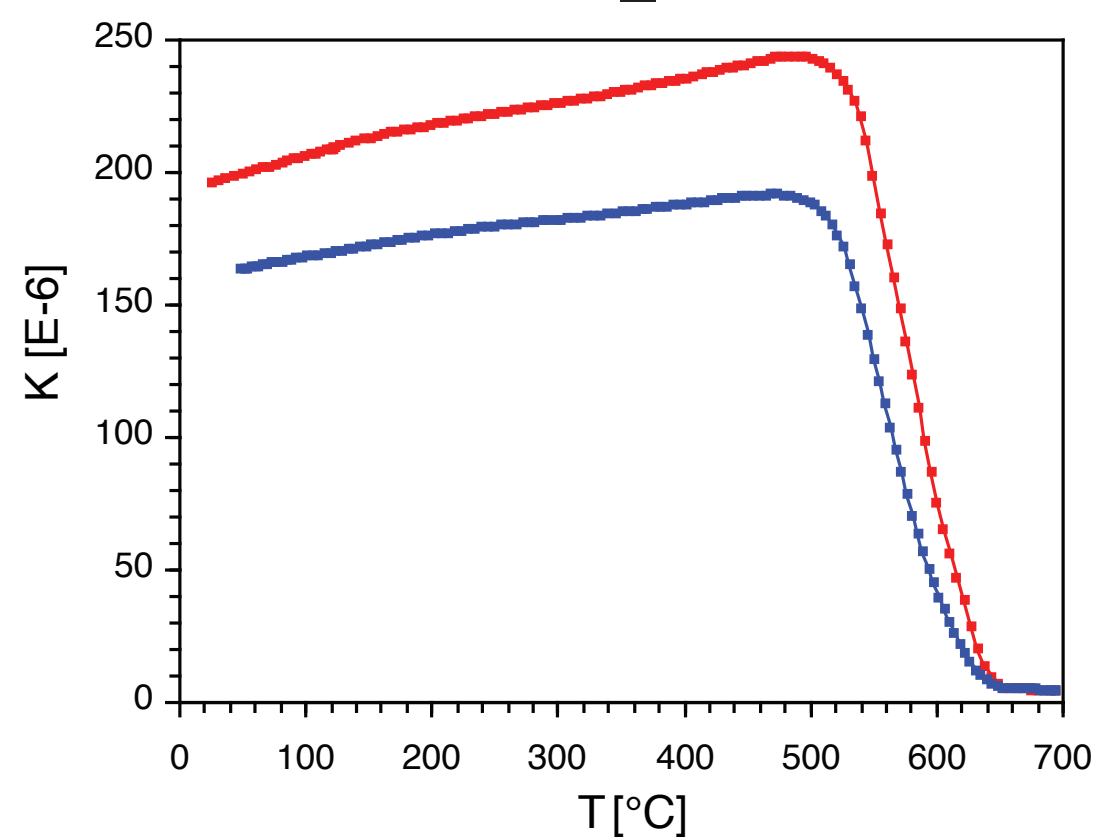

b)

PT\_D

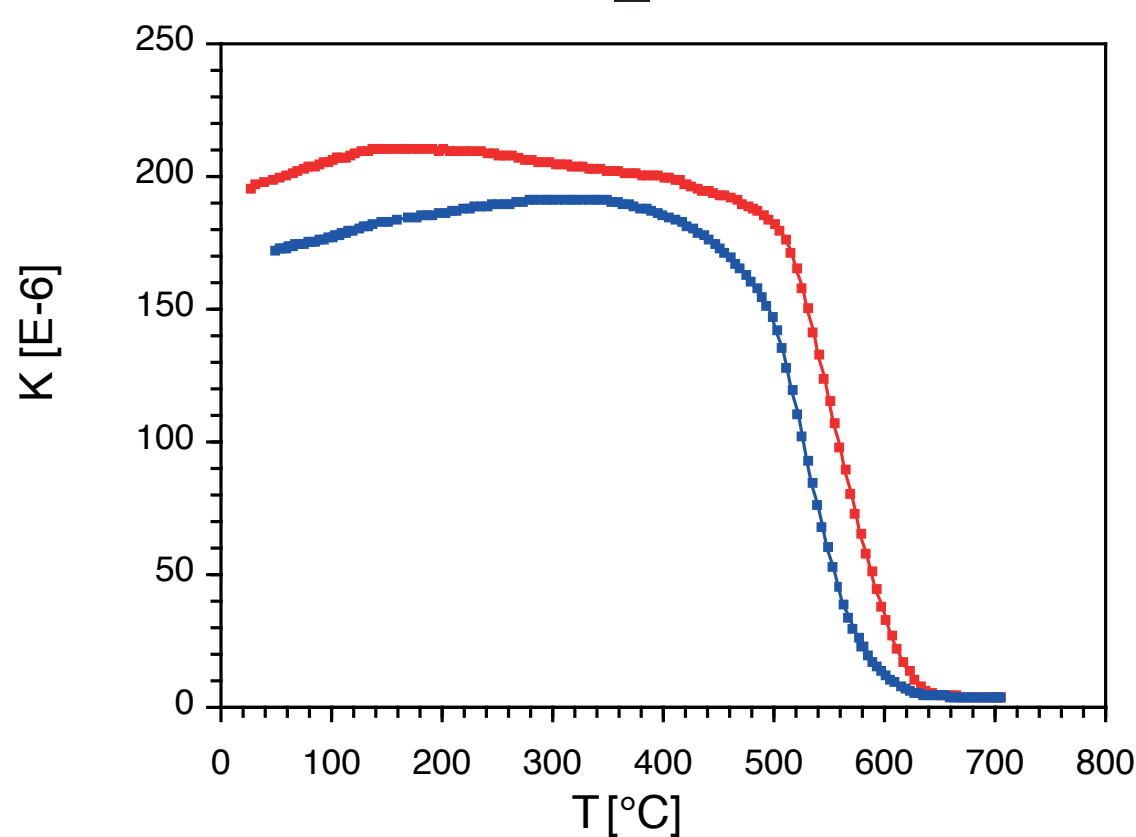

Supplement: S1 Fig — (PDF) [file pone.0351625.s001.pdf]

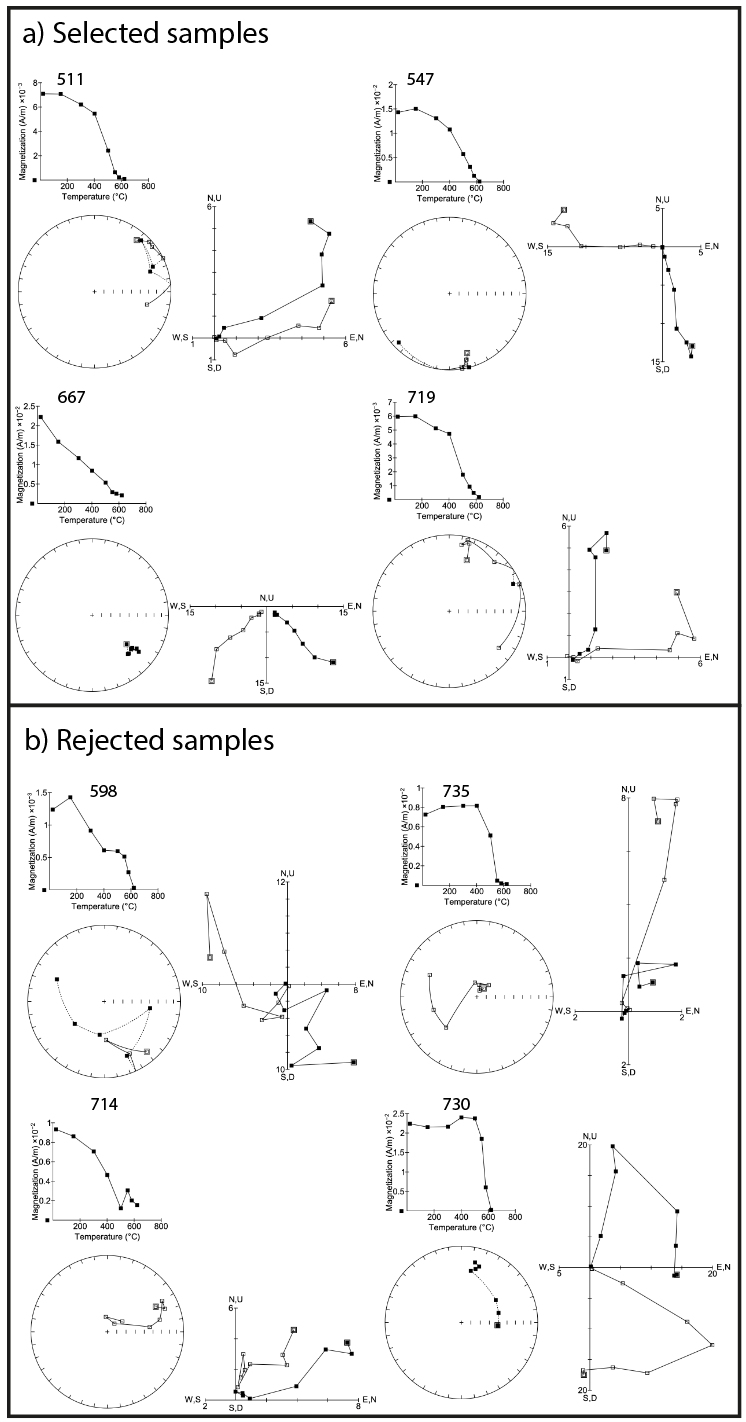

Supplement: S2 Fig — (PNG) [file pone.0351625.s002.png]

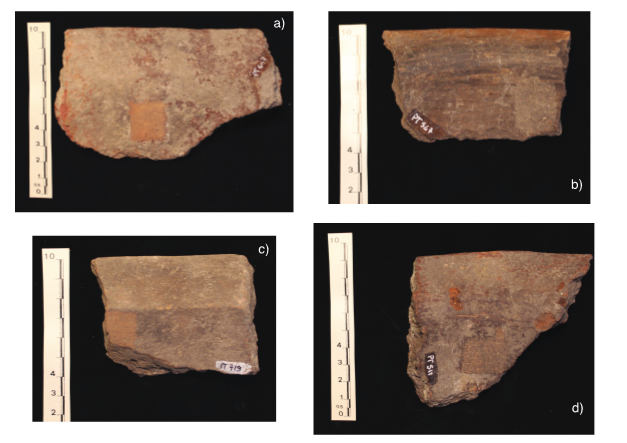

Supplement: S3 Fig — (PNG) [file pone.0351625.s003.png]

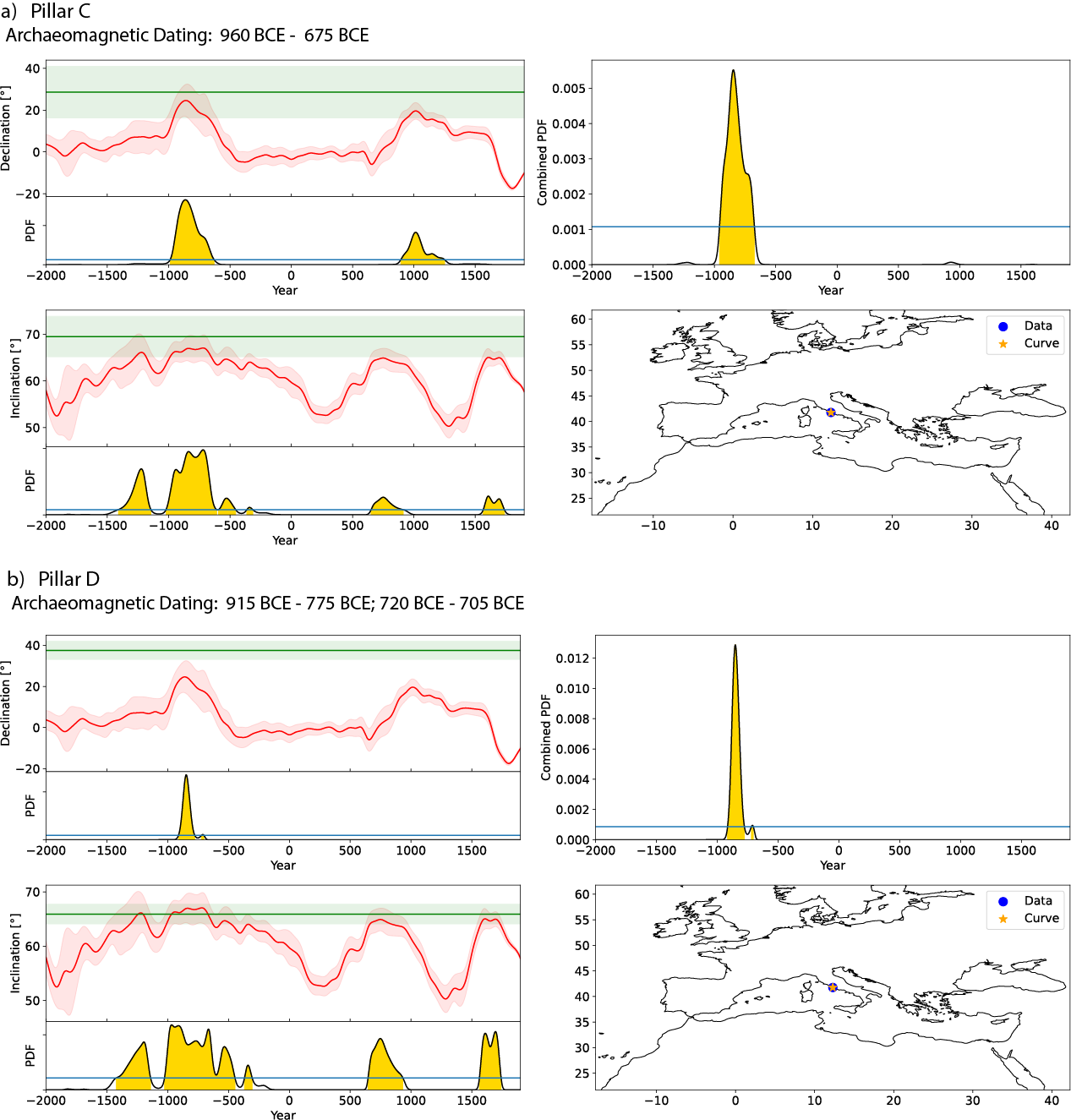

Supplement: S4 Fig — (PNG) [file pone.0351625.s004.png]
